# A quantitative study on the impact of educational modules on the awareness of postural ergonomics among the dental clinical trainees of Jouf University: A North Saudi Arabian Cohort

**DOI:** 10.1016/j.heliyon.2024.e24090

**Published:** 2024-01-04

**Authors:** Merin Mathew, Radhika Doppalapudi, Jothish Ravi

**Affiliations:** a Department of Prosthetic Dental Sciences, Jouf University, Saudi Arabia; b Department of Oral Medicine & Radiology, Jouf University, Saudi Arabia; c Department of Restorative and Endodontics, Jouf University, Saudi Arabia

**Keywords:** Ergonomic education, Posture ergonomics, Musculoskeletal pain, Clinical productivity

## Abstract

This research aimed to evaluate the difference between self-reported and actual work postures among dental clinical trainees and the effect of health education on their ergonomic postures. Bad postures induce fatigue, musculoskeletal pain, errors, a negative attitude, and job dissatisfaction. It is necessary to evaluate the awareness of posture ergonomics among clinical trainees as the bad postures captured during their learning years transform into habits that could affect future productivity. Hence, a three-level quantitative study was conducted among the dental trainees at Jouf University, Saudi Arabia. The first level comprised a self-reported survey and an observational study to record the working postures of the participants. At the second level, appropriate health education is customized based on the findings observed at the first level. An unannounced observational study was conducted at the third level to assess the impact of ergonomic education on the working postures of the participants. Gender and the academic year level of the participants were the variables considered in the study. The study found that the participants overrated their correct postures (P = 0.005). Fortunately, the final post-ergonomic education observations found a significant improvement in work posture (8.6 vs 12.4), regardless of the variables considered in the study (P = 0.001). The three best correct postures were placing the feet resting on the floor (52.6 vs 92.8 %), using the seat of a dental chair (57.9 vs 89.5 %), and using an upright position of the legs at the thigh-to-leg angle (53.9 % vs 86.2 %). Therefore, the study emphasizes the importance of training in ergonomics and regular, periodic observation to eradicate bad postures before they become a habit among young dentists.

## 1 Introduction

The dental practice involves various labour-intensive clinical and laboratory procedures that require great precision, attention, and patience [1]. For the dentist and his team to do their best work and for the dental practice to be successful in the long run, they need the right working conditions [2]. Dental care professionals thus need high mental and physical efficacy to maintain ergonomic positions over a long period of time [1].

The ergonomics attributed to dentistry primarily target the dental practitioner, followed by the team involved. In four-handed dentistry, when the dentists and dental assistants work together, the ideal work posture allows the dentist to perform clinical procedures on patients with significant physical and psychological comfort. Also, a proper posture enables the dentist to work more efficiently and with higher precision throughout the day. A wrong posture may induce fatigue, musculoskeletal pain, an increased chance of errors, and a negative attitude and environment at the workplace [3].

Several studies on the prevalence of musculoskeletal disorders attributed to bad working posture among dentists [[4], [5], [6], [7], [8], [9]] “The prevalence of musculoskeletal pain among dentists working in various parts of Saudi Arabia was reported in a few studies as 70 % in Jeddah [4], 90 % in Riyadh [5], and 77.9 % in Hail" [6]. All these studies were on self-reported work postures and musculoskeletal symptoms. However, to the best of our knowledge, no study reported the use of the observational method for recording work postures or the subsequent impact of health education interventions on the existing work postures of dentists. Moreover, awareness, training, and adoption of ergonomic work posture are essential for a successful dental practice. Good postures should be taught, trained, and implemented at the beginning of dental careers to establish ergonomic practices. In this context, dental students need education on ergonomics to continue practicing efficiently throughout their careers. Therefore, the present study aimed to assess the working postures of students in the College of Dentistry at Jouf University. The participants were then educated about ergonomics and evaluated to assess the impact of that training.

## 2 Materials and methods

### 2.1 Study design, setting, and participants

A three-level quantitative study was conducted based on the self-reported survey and observational findings after the ergonomic health education. The first level included an observational study to record the working postures of the participants, followed by a self-reported survey about working postures. In the second level, after 15 days of the first level, appropriate health education is tailored based on the findings observed in the first level. In the third level, an observational study was performed after 15 days to assess the impact of ergonomic education on changing work postures among the participants. All third-year to sixth-year (internship) dentistry students working in the clinical setting at the College of Dentistry, Jouf University, Saudi Arabia, were invited to participate in this study. Those who volunteered their informed consent to participate were included in the study without further sampling.

### 2.2 Sample size

The sample size was calculated based on the fact that changes in the prevalence of non-ergonomic postures were reported by Dalia et al. in Saudi Arabia. Considering a 95 % confidence interval (CI) and 80 % power, 142 individuals were sufficient to detect a clinically significant difference of 10 %. Therefore, the total sample size was adjusted to 150, expecting a 5 % chance of attrition [2].

### 2.3 Data collection

**Fig. 1:**
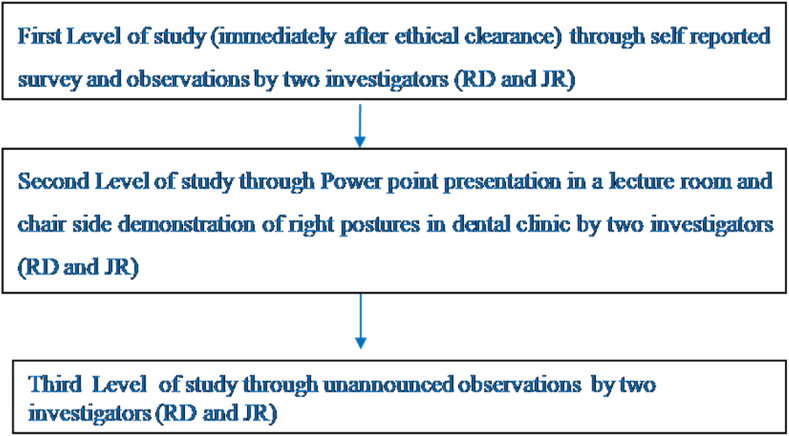
Flowchart of methodology.

Data was collected using a pretested questionnaire and direct observation (Fig. 1).

#### 2.3.1 Questionnaire

A questionnaire consisting of two parts was developed for this study. The first part of the questionnaire included participants' demographic data. The second part included the 14-item compliance assessment of dental ergonomic posture requirements (CADEP), initially developed by Garcia et al. [10]. Four expert dental faculties evaluated the content and factual validity of the questionnaire, and all fourteen items were essential to include in the questionnaire. The validity of the questionnaire was tested with Cronbach's alpha {α = 0.88}. The questionnaire was distributed among the participants, and the responses were collected on the same day. The same questionnaire items were used to record observational data.

#### 2.3.2 Observational data

Two investigators were responsible (authors 2 and 3) for collecting observational data regarding the actual ergonomic postures of the participants in the clinical settings. Training and calibration of the examiners were conducted before the data collection; the intra-examiner reliabilities were 0.92 and 0.94, and the inter-examiner reliability was 0.90.

The working postures of the participants were evaluated during their comprehensive clinic sessions. Each participant's working posture was considered a study variable, and working postures were recorded by direct observation by the evaluators (authors 2 and 3) who were physically present in the workplace at a distance of 6–8 feet from the operator. The operator's ergonomic postures were recorded 10 min after the beginning of his clinical activity, allowing the operators to settle down with their common postures. Participants were unaware of the evaluator recording their working postures to limit or avoid the “Hawthorne Effect."

### 2.4 Ergonomic education

After the first phase of data collection, 2 h of ergonomic education were provided with the help of PowerPoint presentations for 1 h, followed by clinical chair-side demonstrations of appropriate ergonomic postures and bad postures for 1 h by a senior faculty member who was not a part of this research.

### 2.5 Ethical considerations

The Local Committee of Bioethics (LCBE) approved the study design and data collection at Jouf University (Ref. # 5-02-43). All participants knew the study objectives were to ensure complete privacy and confidentiality. Furthermore, participants were advised that they might leave the study at any moment without repercussions.

### 2.6 Statistical analysis

Statistical analysis was performed using Statistical Software for the Social Sciences, version 20 [IBM Corp., Armonk, United States]. First, descriptive statistical analysis was calculated, and the statistical significance of relationships between variables was examined. The participants' age, gender, and level of study were considered independent variables in this study. The data's normality was examined using the Kolmogorov-Smirnov (KS) test. Chi-square for categorical data and either a paired samples't’ test or a two-way ANOVA were used for before and after comparisons of quantitative data.

## 3 Results

### 3.1 Demographic characteristics of participants

Although 155 people volunteered to participate in the study, data from three participants had to be omitted from the final analysis as they were not available for recording postures in clinical settings. Hence, the final analysis considered only 152 participant responses. Males made up 93 of the 152 participants. Forty-eight participants were from the third, 40 from the fourth, 37 from the fifth, and 27 from the sixth (internship) academic year. The majority, 139 out of 152, were between the ages of 21 and 30, as represented in Table 1.

**Table 1:** Demographic Characteristics of participants.

Item	Males	Females	Whole group
**Academic year**			
3rd year	28(30.1 %)	20(33.9 %)	48(31.6 %)
4th year	26(28 %)	14(23.7 %)	40(26.3 %)
5th year	23(24.7 %)	14(23.7 %)	37(24.3 %)
Intern	16(17.2 %)	11(18.6 %)	27(17.8 %)
Total	93(100 %)	59(100 %)	152(100 %)
**Age group**			
≤20 years	04(4.3 %)	06(10.2 %)	10(6.6 %)
21–30 years	86(92.5 %)	53(89.8 %)	139(91.4 %)
>30 years	03(3.2 %)	00	03(2 %)

### 3.2 Comparison of ergonomic postures of self-reported and an observational method

Table 2 compares the self-reported ergonomic postures with the observed postures before conducting health education. There is a statistically significant difference between the participants' self-reported and actual working postures (p = 0.005). Participants' self-reported results were higher than the basic postures that investigators recorded by direct observation.

**Table 2:** Comparison of ergonomic postures of self-reported and an observational method.

SL NO	ITEM	Percentage of responses with right ergonomic posture	
		Self-reported	Observed
1	Legs in the upright position (thigh/leg angle)	120(78.9 %)	82(53.9 %)
2	Feet resting on the floor	125(82.2 %)	80(52.6 %)
3	Thighs in a horizontal position (angle between the thighs)	112(73.7 %)	71(46.7 %)
4	Tilting of the spine	102(67.1 %)	67(44.1 %)
5	Spine concerning lumbar support	140(92.1 %)	72(47.4 %)
6	Use of the seat of a dental stool	152(100 %)	88(57.9 %)
7	The patient positioned in the patient chair	132(86.8 %)	74(48.7 %)
8	Position of the headrest of the patient chair	110(72.4 %)	90(59.2 %)
9	Seat height concerning the leg of the operator located under the backrest	112(73.7 %)	72(47.7 %)
10	Dental operator light	123(80.9 %)	86(56.6 %)
11	Distance between patient's mouth and operator's eyes	108(71.1 %)	91(59.9 %)
12	Working arm	110(72.4 %)	70(46.1 %)
13	Supporting arm	98(64.5 %)	67(44.1 %)
14	Position of hand instrument used to perform clinical procedures	102(67.1 %)	74(48.7 %)

Chi-square test, χ^2^ = 18.4 df = 6, P value = 0.005*

### 3.3 Comparison of compliance assessment of dental ergonomic postures

Table 3 compares the various aspects of ideal ergonomic postures among male and female participants. Significant improvements in work posture were observed after ergonomic education. Male participants showed greater item improvement than their female counterparts. The top three correctly followed aspects of ergonomic posture after health education were feet resting on the floor (52.6 vs 92.8 %), use of the seat of a dental stool (57.9 vs 89.5 %), and the legs' upright position considered with a thigh-to-leg angle (53.9 % vs 86.2 %). The bottom three rightly followed ergonomic aspects were spine concerning lumbar support (67.1 vs 47.4 %), tilting of the spine (72.4 vs 44.1 %), and working arm position (72.4 vs 50.8 %).

**Table 3:** Comparison of Compliance of ergonomic postures before and after education.

ITEM		Percentage of clinical encounters with right ergonomic posture					
		Before			After		
		Male	Female	Total	Male	Female	Total
1	Legs in an upright position (thigh/leg angle)	45(48.4 %)	37(62.7 %)	82(53.9 %)	87(93.5 %)	44(74.6 %)	131(86.2 %)
2	Feet resting on the floor	48(51.6 %)	32(54.2 %)	80(52.6 %)	90(96.8 %)	51(86.4 %)	141(92.8 %)
3	Thighs in a horizontal position (angle between the thighs)	47(50.5 %)	24(40.7 %)	71(46.7 %)	76(81.7 %)	48(81.4 %)	124(81.6 %)
4	Tilting of the spine	44(47.3 %)	23(39 %)	67(44.1 %)	60(64.5 %)	50(84.7 %)	110(72.4 %)
5	Spine concerning lumbar support	42(45.2 %)	30(50.8 %)	72(47.4 %)	55(59.1 %)	47(79.7 %)	102(67.1 %)
6	Use of the seat of a dental stool	55(59.1 %)	33(55.9 %)	88(57.9 %)	86(92.5 %)	40(67.8 %)	126(89.5 %)
7	The patient positioned in the patient chair	49(52.7 %)	25(42.4 %)	74(48.7 %)	81(87.1 %)	39(66.1 %)	120(78.9 %)
8	Position of the headrest of the patient chair	59(63.4 %)	31(52.5 %)	90(59.2 %)	83(89.2 %)	42(71.2 %)	125(82.2 %)
9	Seat height in relation to the leg of the operator located under the backrest	46(49.5 %)	26(44.1 %)	72(47.4 %)	73(78.5 %)	46(78 %)	119(78.3 %)
10	Dental operator light	52(55.9 %)	34(57.6 %)	86(56.6 %)	79(84.9 %)	49(83.1 %)	128(84.2 %)
11	Distance between patient's mouth and operator's eyes	53(57 %)	38(64.4 %)	91(59.9 %)	88(94.6 %)	42(72.9 %)	130(86 %)
12	Working arm	40(43 %)	30(50.8 %)	70(46.1 %)	73(78.5 %)	37(62.7 %)	110(72.4 %)
13	Supporting arm	42(45.2 %)	25(42.4 %)	67(44.1 %)	80(86 %)	35(59.3 %)	115(75.7 %)
14	Position of hand instrument used to perform clinical procedures	45(48.4 %)	29(49.2 %)	74(48.7 %)	79(84.9 %)	43(72.9 %)	122(80.3 %)

Chi-square test, χ2 = 12.96, df = 6, P value = 0.002

### 3.4 Comparison of observed compliance assessments of dental ergonomic postures

Table 4 compares ergonomic postures with the academic year of the participants. Participants who belonged to the internship and fourth academic year were more compliant with ergonomic postures than other groups.

**Table 4:** Comparison of observed Compliance assessment of dental ergonomic postures.

ITEM No.	Percentage of clinical encounters with correct ergonomic posture							
	Before				After			
	3rd year	4th Year	5th year	Intern	3rd year	4th Year	5th year	Intern
1	23(47.9 %)	23(57.5 %)	18(48.6 %)	18(66.7 %)	38(79.2 %)	34(85 %)	35(94.6 %)	24(88.9 %)
2	18(37.5 %)	20(50 %)	24(64.9 %)	18(66.7 %)	43(89.6 %)	37(92.5 %)	36(97.3 %)	25(92.6 %)
3	16(33.3 %)	18(45 %)	16(43.2 %)	21(77.8 %)	39(81.3 %)	31(77.5 %)	33(89.2 %)	21(77.8 %)
4	16(33.3 %)	15(37.5 %)	18(48.6 %)	18(66.7 %)	36(75 %)	21(52.5 %)	30(81.1 %)	23(85.2 %)
5	18(37.5 %)	16(40 %)	21(56.8 %)	17(63 %)	28(58.3 %)	21(52.5 %)	31(83.8 %)	22(81.5 %)
6	29(60.4 %)	20(50 %)	18(48.6 %)	21(77.8 %)	38(79.2 %)	33(82.5 %)	33(89.2 %)	22(81.5 %)
7	17(35.4 %)	18(45 %)	23(62.2 %)	16(59.3 %)	32(66.7 %)	28(70 %)	35(94.6 %)	25(92.6 %)
8	28(58.3 %)	28(70 %)	18(48.6 %)	16(59.3 %)	38(79.2 %)	34(85 %)	29(78.4 %)	24(88.9 %)
9	20(41.7 %)	16(40 %)	25(67.6 %)	13(48.1 %)	35(72.9 %)	33(82.5 %)	31(83.4 %)	20(74.1 %)
10	19(39.6 %)	24(60 %)	22(59.5 %)	21(77.8 %)	44(91.7 %)	29(72.5 %)	34(91.9 %)	21(77.8 %)
11	30(62.5 %)	22(55 %)	23(62.2 %)	16(59.3 %)	39(81.3 %)	35(87.5 %)	35(94.6 %)	22(81.5 %)
12	21(43.8 %)	16(40 %)	23(62.2 %)	10(37 %)	34(70.8 %)	27(67.5 %)	25(67.6 %)	24(88.9 %)
13	17(35.4 %)	20(50 %)	19(51.4 %)	11(40.7 %)	34(70.8 %)	30(75 %)	30(81.1 %)	21(77.8 %)
14	20(41.7 %)	20(50 %)	21(56.8 %)	13(48.1 %)	36(75 %)	32(80 %)	34(91.9 %)	20(74.1 %)

### 3.5 Comparison of observed mean compliance scores with variables

Each participant scored on a 14-item scale. The correct position of each item was scored as “1," and inappropriate work positions were scored as “0." As described in Table 5, there was no statistically significant association between gender and academic year, and the only statistically significant difference (P = 0.001) found was before and after health education (8.6 vs 12.4).

**Table 5:** Comparison of observed mean compliance scores with variables.

	Mean ± SD		Statistical test	*P*-Value
	Before	After		
GENDER				
Male	8.4 ± 1.2	12.1 ± 1.8	Paired ‘*t*-test	0.078
Female	8.8 ± 2	11 ± 2.2		0.272
ACADEMIC YEAR			Two-way ANOVA	
3rd year	8.2 ± 1.8	10.4 ± 2.2		0.326
4th year	9.4 ± 2.4	13.1 ± 2.1		
5th year	8.4 ± 2	11 ± 1.4		
Intern	9 ± 1.6	12.8 ± 1.8		
OVERALL	8.6 ± 2.1	12.4 ± 4.6	Paired ‘*t*-test	0.001

## 4 Discussion

Musculoskeletal pain and disorders (MSD) are widespread among dentists, with several factors increasing their prevalence. As reported by researchers [[1], [2], [3], [4], [5], [6], [7], [8], [9]], identifying the causes of these disorders is essential as it can decrease the chances of skeletal disorders and associated problems. In addition, good posture during work helps to reduce energy expenditure, thereby improving function, comfort, and quality of work [[11], [12], [13]]. The present study thus evaluated the self-reported and observed ergonomic postures before and after ergonomic education among dental undergraduate students and interns who work in dental clinics. The research background in Table 1 demonstrates the demographic data in the study: more male participants than females and most participants were between 20 and 30.

The significant difference between the self-reported and observed values of ergonomic postures shown in Table 2 indicates that the participants did not maintain the correct working positions during the practice, even if they had reported incorrect positions. During the five-year bachelor dental surgery course at Jouf University, the detailed ergonomics aspect is taught in the preclinical skill course in the second year. However, when they begin their actual clinical practice, the majority of the students neglect the importance of balanced postures. The observations revealed that the participants' working postures improved significantly after the ergonomic education sessions. Similar results were obtained from a study conducted among healthcare professionals in the eastern province of Saudi Arabia [14]. The consequences of inappropriate work posture are often chronic, cumulative, and severe. They reduce the clinician's productivity and affect the quality of his life. Most clinicians ignore the signs until they become very conspicuous. Therefore, frequent rechecks on proper work posture and positions are desirable among dental students. The timely correction would reduce energy expenditure and prevent the development of physical ailments that are otherwise considered occupational hazards in dentistry [15].

Ergonomic education was thus provided in the present study after analyzing the self-reported and observed ergonomic positions. The ergonomics standard ISO 11226 describes a balanced or neutral position for the dentist. Maintaining this stance within the constraints allowed by practicing settings during all phases of clinical activities is advised. This natural, stress-free, and symmetrical sitting posture enables the human body's locomotor physiology. The following are the conditions for a balanced posture: “a straight back with a maximum forward inclination of the trunk of 20°, a maximum forward inclination of the head of 20–25° from the trunk, arms positioned along the body and forward-oriented within 10°, forearms raised maximum 25° from the horizontal line, the angle between thighs and shanks of 105–110° or more, thighs separated up to 45°, and shanks perpendicular to the floor or slightly posterior. “The feet on the floor are facing forward in the same plane as the shanks and are positioned symmetrically under the operator's hands" [1]. The above-mentioned balanced position presumes minimal contractions and muscular tensions in the body. A person may obtain the best ergonomic posture during dental practice by sitting upright and transferring body weight along the spinal axis to the dental chair base. The dentist's lower back should contact the chair's back for lumbar support and a straight-back posture. Body stability and transfer of body weight to the soles of the feet can be achieved by positioning the feet flat on the ground and maintaining a 90°–110° angle between the thigh and lower leg. This position reduces lower back muscle compression, and the pain associated with the neck, vertebra, and back can be managed [[16], [17], [18]]. To further reduce the likelihood of developing job-related muscular and skeletal disorders, a seated posture should not be selected as the single and permanent working position, but rather a diverse and dynamic working method can be adopted in daily work [[19], [20], [21], [22]].

It is reported that the students need help putting theory into practice because they ignore the body postures during the activities. Also, no significant association was found between gender differences and musculoskeletal disorders [23,24]. Remarkable progress was observed in the current study regarding the participants' postures after ergonomic training, as described in Table 3. Both male and female students exhibited significant improvement in their ergonomics. Notable advancement was observed in males rather than females.

On comparing the level of study of the participants and their work postures, as shown in Table 4, it is observed that the interns and fourth-year students were more organized and maintained balanced ergonomics compared to another academic batch. Third-year students showed maximum improvements in their ergonomics after ergonomic training. However, no statistically significant relationship between the independent variable, the level of study of the participant, and the optimal ergonomic position could be found. Therefore, it is essential to apply theory to everyday clinical practice. It would be challenging to alter the acquired habit later. Therefore, if the students adopt a neutral posture from the beginning of their clinical practice, future musculoskeletal problems could be avoided [25].

Therefore, ergonomic training courses with theoretical and practical modules in the dental academic curriculum could enhance the implementation of adequately balanced ergonomic positions at the beginning of the dental career [25]. This is in agreement with the present study as described in Table 5; significant improvement in the proper ergonomic posture among participants was noticed after ergonomic education, suggesting the need for periodic workshops and awareness programs in the field of proper work postures in dentistry.

The chosen participants are the archetype of future dentists in Saudi Arabia. Musculoskeletal problems are prevalent among Saudi dentists. Evaluation of their theoretical knowledge about correct working postures and the actual practice conditions they follow could be a guide for understanding the primary factor that causes musculoskeletal disorders among dentists in Saudi Arabia. Most of the participants in the current study got the benefit of an awareness program conducted regarding dental ergonomics.

The present study measured only the percentage of participants in a specific position, and the time percentage in a specific position is not included as an objective for evaluation in this study. Additionally, it is important to observe ergonomic postures in real life practice, as they may slightly vary from dental teaching clinical settings.

## 5 Conclusions

The current findings indicate the significant difference between self-reported and observed values of correct dental ergonomics, suggesting the need for adequate guidance to implement the theoretical knowledge into clinical practice. Follow-up with the participants after health education revealed the impact of motivation and regular reminders or awareness programs to adopt correct ergonomic postures during their clinical training.

## Funding statement

10.13039/501100007614Jouf University funded this research, Vice Rectorate for Graduate Studies and Scientific Research, Deanship of Scientific Research (DSR), grant number: DSR-2021-01-0118.

## Additional information

No additional information is available for this paper.

## CRediT authorship contribution statement

**Merin Mathew:** Conceptualization, Data curation, Formal analysis, Funding acquisition, Investigation, Project administration, Resources, Software, Supervision, Validation, Visualization, Writing – original draft, Writing – review & editing. **Radhika Doppalapudi:** Conceptualization, Investigation, Methodology, Writing – review & editing. **Jothish Ravi:** Conceptualization, Investigation, Methodology, Writing – review & editing.

## Declaration of competing interest

The authors declare that they have no known competing financial interests or personal relationships that could have appeared to influence the work reported in this paper.

## References

[1] Pirvu C., Patrascu I., Pirvu D., Ionescu C. (2014). The dentist's operating posture – ergonomic aspects. Journal of Medicine and Life.

[2] Beibei F., Qi L., Yuling W. (2014). Prevalence of work-related musculoskeletal symptoms of the neck and upper extremity among dentists in China. BMJ Open.

[3] Ratzon N., Yaros M., Mizlik A., Kanner T. (2000). Musculoskeletal symptoms among dentists concerning work posture. Work.

[4] Dalia E.M., Nujud S.A., Ahmad A.S., Mohammed Y.A. (2019). Prevalence of work-related musculoskeletal disorders and ergonomic practice among dentists in Jeddah, Saudi Arabia. ClinCosmetInvestig Dent.

[5] Omar A.A., Nouf S.A., Waad M.A., Emad M.M., Hind S.A. (2016). Prevalence of musculoskeletal pain of the neck, upper extremities and lower back among dental practitioners working in Riyadh, Saudi Arabia: a cross-sectional study. BMJ Open.

[6] Mohammad A., Sameer S., Ammar A.S., Moazzy M., Syed S.H. (2015). Prevalence of musculoskeletal disorders among dentists in the Hail Region of Saudi Arabia. Ann. Saudi Med..

[7] Rambabu T., Suneetha K. (2014). Prevalence of work-related musculoskeletal disorders among physicians, surgeons, and dentists: a comparative study. Ann. Med. Health Sci. Res..

[8] Haas Y., Naser A., Haenel J., Fraeulin L., Holzgreve F., Erbe C. (2020). Prevalence of self-reported musculoskeletal disorders of the hand and associated conducted therapy approaches among dentists and dental assistants in Germany. PLoS One.

[9] Kumar M., Pai K.M., Vineetha R. (2020). Occupation-related musculoskeletal disorders among dental professionals. Medicine and Pharmacy Reports.

[10] Garcia P.P., Wajngarten D., Campos J.A. (2018). Development of a method to assess compliance with ergonomic posture in dental students. J. Educ. Health Promot..

[11] Gupta A., Ankola A.V., Hebbal M. (2013). Dental ergonomics to combat musculoskeletal disorders: a review. International journal of occupational safety and ergonomics. JOSE..

[12] Aljanakh M., Shaikh S., Siddiqui A.A., Al-Mansour M., Hassan S.S. (2015). Prevalence of musculoskeletal disorders among dentists in the Ha’il Region of Saudi Arabia. Ann. Saudi Med..

[13] Haslegrave C.M. (1994). What do we mean by a 'working posture'?. Ergonomics.

[14] Sukainah S., Hazim A.L., Sultan T., Al-Otaibi Nawal H.H. (2022). Knowledge, attitudes, and practices regarding ergonomic hazards among healthcare workers in a Saudi government hospital. J. Multidiscip. Healthc..

[15] Chenna D., Pentapati K.C., Kumar M., Madi M., Siddiq H. (2022). Prevalence of musculoskeletal disorders among dental healthcare providers: a systematic review and meta-analysis. F1000Research.

[16] Pungwattana V., Rodanant P. (2021). Ergonomic working posture in dentistry: importance of body and limb dimensions. Mo. Dent. J..

[17] Garcia P.P., Campos J.A. (2013). Risk of musculoskeletal disorders in upper limbs in dental students: concordance of different body angle estimation methods. Indian J. Dent. Res.: official publication of Indian Society for Dental Research.

[18] Valachi B., Valachi K. (2003). Preventing musculoskeletal disorders in clinical dentistry: strategies to address the mechanisms leading to musculoskeletal disorders. JADA (J. Am. Dent. Assoc.).

[19] Huppert F., Betz W., Maurer-Grubinger C., Holzgreve F., Fraeulin L., Filmann N., Groneberg D.A., Ohlendorf D. (2021). Influence of design of dentist's chairs on body posture for dentists with different working experience. BMC Muscoskel. Disord..

[20] Pope-Ford R., Pope-Ozimba J. (2020). Musculoskeletal disorders and emergent themes of psychosocial factors and their impact on health in dentistry. Work.

[21] Kierklo A., Kobus A., Jaworska M., Botuliński B. (2011). Work-related musculoskeletal disorders among dentists - a questionnaire survey. Ann. Agric. Environ. Med.: AAEM..

[22] Gandavadi A., Ramsay J.R., Burke F.J. (2007). Assessment of dental student posture in two seating conditions using RULA methodology - a pilot study. Br. Dent. J..

[23] Corrocher P.A., Presoto C.D., Campos J.A.D.B., Garcia P.P.N.S. (2014). The association between restorative preclinical activities and musculoskeletal disorders. Eur. J. Dent. Educ..

[24] Treaster D.E., Burr D. (2004). Gender differences in the prevalence of upper extremity musculoskeletal disorders. Ergonomics.

[25] Movahhed T., Dehghani M., Arghami S., Arghami A. (2016). Do dental students have a neutral working posture?. J. Back Musculoskelet. Rehabil..

