# Supplementary material for: A quantitative study on the impact of educational modules on the awareness of postural ergonomics among the dental clinical trainees of Jouf University: A North Saudi Arabian Cohort
Source: Heliyon. 2024 Jan 4;10(1):e24090. doi: 10.1016/j.heliyon.2024.e24090 (PMC10789623; doi:10.1016/j.heliyon.2024.e24090)
Supplement: Multimedia component 1 [file mmc1.docx]

**A quantitative study on the impact of educational modules on the awareness of postural ergonomics among the dental clinical trainees of Jouf University: A North Saudi Arabian Cohort**

Dear Participant, Please mark your response to the following questions

**Part I: Background details**

1. Age……………. Years
2. Gender: Male [ ] Female [ ]
3. You are a ………

Intern [ ]

5^th^ academic year student [ ]

4^th^ academic year student [ ]

3^rd^ academic year student [ ]

1. Have you ever heard about ergonomics in dentistry?

Yes [ ] No [ ]

**Part II: Questionnaire related to Dental Ergonomic Posture**

**1. Legs in an upright position (thighs/leg angles)**

( )^1^ less than 90° ( ) ^2^ equal to 90°( ) ^3^ greater than 90°

**2. Feet rest on the floor**

( )^1^ both feet flat on the floor ( ) ^2^ only one foot flat on the floor ( ) ^3^ both feet on the stool

**3. Thighs in horizontal position (angle between thighs)**

( )^1^equal to 90° ( )^2^ equal to zero (parallel) ( )^3^equal to 70°

**4. Tilting of the spine**

( )^1^posterior position ( )^2^ anterior position ( )^3^ middle position

( )^4^posterior position tilted to the right ( )^5^ posterior position tilted to the left

( )^6^ anterior position tilted to the right ( )^7^ anterior position tilted to the right

( )^8^ middle position tilted to the right ( )^9^ middle position tilted to the left

**5. Spine in relation to lumbar support**

( )^1^ support on the back of the chair ( )^2^ no support on the back of the chair

**6. Using the seat of the dentist's chair**

( )^1^ occupied the entire seat of the chair ( )^2^ did not occupy the entire seat of the chair

**7. Patient position in the patient chair**

( )^1^ Leaning back with mouth at knee level ( )^2^ leaning back with knee over mouth

( )^3^ half lying

**8. Position of the headrest of the patient chair**

( )^1^ on the longitudinal axis when examining the upper or lower anterior teeth

( )^2^ tilted forward working on the jaw ( )^3^ tilted back working on the jaw

( )^4^ on the long‑axis working on the jaw ( )^5^ tilted forward working on the maxilla

( )^6^ tilted back working on the maxilla( )^7^ on the long‑axis working on the maxilla

**9. Seat height in relation to the operator's leg, which is located under the backrest**

( )^1^ thigh/leg without pressure of dental chair

( )^2^ thigh/leg with pressure of dental chair

**10. Dental operator light**

( )^1^ at the head of the patient for work on themaxilla

( )^2^ perpendicular to the patient’s head for work on the jaw

( )^3^ without respect to the work area

**11. Distance between patient’s mouth and operator’s eyes**

( )^1^ 30 to 40 cm ( )^2^ 2<30 cm ( )^3^ 3>40 cm

**12. Working arm:** ( )^1^ next to the body ( )^2^ partially raised ( )^3^ fully raised

**13. Supporting arm**

( )^1^ next to the body ( )^2^ partially raised ( )^3^ fully raised

( )^4^ embracing the patient’s head ( )^5^ raised to support the chair

**14. Placement of hand instruments used to perform clinical procedures**

( )^1^ ideal space to be achieved ( )^2^ maximum space to be achieved

( )^3^ outside the space to be achieved
